# Toward a politics of shame: cripping understandings of affect in disabled people’s encounters with unsolicited advice

**DOI:** 10.3389/fsoc.2025.1401812

**Published:** 2025-03-19

**Authors:** Megan Ingram

**Affiliations:** School of Kinesiology and Health Studies, Queen’s University, Kingston, ON, Canada

**Keywords:** disability, unsolicited advice, emotion, affect, shame, resistance, blame, apathy

## Abstract

The prevalence of unsolicited advice in the lives of disabled people is well-catalogued in the mass of articles and social media posts dedicated to the issue. However, less is known about the affective impacts of this advice on disabled people and the potential resistance that may be enacted, such as shame, toward affects labelled negative. The present manuscript builds from original qualitative research to explore the links between emotion, mind, and body that occur in interactions involving unsolicited advice between disabled and non-disabled individuals. Non-probability convenience sampling was used to recruit 15 disabled individuals in Ontario, Canada for participation in semi-structured qualitative interviews that were inductively coded and narratively restoried. Building from these narrative accounts, the research addresses (1) the affective impacts of unsolicited advice on disabled people and (2) how disabled people negotiate the emotional impact resulting from unsolicited advice and *blame culture* individually and collectively. Ultimately, this research argues that, while unsolicited advice acts as a method of blaming and shaming that has the potential to structure disabled peoples’ lives, disabled people resist feeling *ashamed* and instead bridge from initial responses of fear and shame toward other emotions such as apathy and sadness in resistant and potentially empowering ways.

## 1 Introduction

The prevalence of unsolicited advice in the lives of disabled^1^ people is well catalogued in the mass of articles and social media posts dedicated to the issue (e.g., Graham, 2011; Blahovec, 2017; Pulrang, 2020). Unsolicited advice is often outlined in posts as coming from well-intentioned desires to help but ultimately positions disabled people as in need of cure or as having caused the circumstances of their disablement through either action or inaction. This is exemplified by chronically ill content creator MB Marshall who, in a 2024 Instagram reel captioned “Things people have actually said to me (as an chronically ill person)” [sic], lists the unsolicited and often contradictory advice they have received. Some of the examples include “I think you can cure that if you go gluten free,” “have you tried positive affirmations?,” “you should lose weight,” and “you should gain weight.” With over 750 comments from other chronically ill and disabled people commiserating over similar experiences, and jokingly suggesting ever more ludicrous ideas mocking unsolicited advice including “summoning ancient eldritch beings,” these interactions are a microcosm reflecting broader dynamics of disability and unsolicited advice. In particular, these examples reflect persistent ableism that aims to make disabled people responsible for their disability (and thus “fixing” it) while simultaneously constructing them as infantile and incapable and thus in need of advice and/or rescue.

Despite the plethora of online anecdotes surrounding disability and unsolicited advice, less is known about the affective impacts of this advice on disabled people and the potential resistance that may be enacted toward negative affects such as shame. In this work, I conceptualize unsolicited advice as advice given without explicit solicitation of, or requests for, guidance and which is largely understood to be unwanted by the recipient. Unsolicited advice may take the form of explicit advice giving (e.g., “you should…”) but can also come across in less explicit discursive terms such as questions (e.g., “have you tried…?”). Research on advice, primarily undertaken in the disciplines of medicine and cross-cultural psychology, has largely focused on solicited advice and the “potential problematic side-effects of social support interactions” (Boutin-Foster, 2005, p. 5). As such, very little is known about the factors leading to unsolicited advice giving in personal relationships despite prior research indicating that unsolicited advice “tends to have more negative effects than receiving solicited advice” (Feng and Magen, 2016, p. 752).

While this prior research on advice offers insight into the potential affective motivations for the *giving* of unsolicited advice, very little is known about the actual affective experiences, emotive consequences, and resistant strategies of disabled people who *receive* such advice (Ingram, 2023). In articulating affective experiences, I conceptualize affect as articulating the same concept as *emotion.* Both terms work to solve the same problem: “that of distinguishing first-person from third-person feeling, and, by extension, feeling that is contained by an identity from feeling that is not” (Ngai, 2005, p. 27). For this reason, I use affect and emotion interchangeably, viewing them as differing intensities of the same structuring of feeling. Ultimately, what is at issue in the giving and receiving of unsolicited advice is the availability of emotional responses to different parties within the interaction. Understanding what emotions and outward affective performances are available within interactions is crucial due to their capacity to indicate the *political horizon*—what is considered politically desirable within a collectivity (Gould, 2009; Kolarova, 2012).

Scholars working at the intersections of disability and affect have indicated that *shame* in particular is an emotion with considerable political power, particularly within the context of disability (Jóhannsdóttir et al., 2021). Disability’s positioning at the heart of the ‘moral economy’—in which moral sentiments interact with broader sociopolitical contexts—shapes the interpersonal contexts and ways in which disabled people show up in the world (Hughes, 2012). Unsolicited advice is one such example of “a moral tool” (Tabin et al., 2019, p. 90) that emerges from this context as a way to respond to the perceived threat that disability poses to the “carefully constructed myth of the ‘able’ body and self which is foundational to a neoliberal social order” (Liddiard and Slater, 2018, p. 3).

Existing research on disabled experience and affect largely focuses on the solely negative impacts of disablism and moral tools (such as unsolicited advice) or, conversely, seeks to tell a positive story about disability pride. Such research not only positions positive and negative emotions as an intractable binary but further positions emotions labelled negative, such as shame, as the unfortunate but inevitable result of deviating from normative ideals in a disablist society (Jóhannsdóttir et al., 2021). While this binary remains dominant, scholars such as Sarah Ahmed and Sianne Ngai have argued for a move away from these dichotomous classifications. This study resisted this binary classification of emotion and instead sought to explore the following questions with attention to the plurality of emotion that can arise in interpersonal interactions: (1) What are the affective impacts of unsolicited advice on disabled individuals? and (2) How do disabled individuals negotiate the emotional impact resulting from unsolicited advice and ‘blame culture’, individually and collectively? To answer these questions, 15 narrative semi-structured interviews were conducted with disabled individuals in Ontario, Canada.

In the following analysis, I first outline the conceptual framework that shapes the theoretical structure of the analysis (section 2), drawing on diverse literature from across disciplines. I then present the methods used, including semi-structured qualitative interviews, and participant demographics (section 3). This is followed by the presentation of the interview data, in context of the conceptual framework (section 4). This discussion traces the timeline of affective response to unsolicited advice, beginning with initial responses such as fear and shame and bridging over time to emotions such as sadness and apathy, which are experienced and deployed in potentially resistant and empowering ways. In the final section, I present a discussion of the findings and my articulation of what they mean for a crip politics of shame; I then conclude with a brief discussion of research beyond binaries.

## 2 Conceptual framework

### 2.1 Face-threatening acts and politeness theory

Extant literature on advice broadly conceptualizes the interpersonal challenges it poses as originating “from its nature as an intrinsically face-threatening act” (Feng and Magen, 2016, p. 752). Goffman defines ‘face’ in his seminal work *Interaction Ritual* as the “positive social value a person effectively claims for” themselves in a particular contact (Goffman, 1967, p. 5). One’s feelings and sense of self become connected to one’s face, emerging in concert with the ways that one perceives and is perceived in social interaction. Crucially, face is claimed. As a socially situated identity, it does not arise naturally but is claimed when one enacts the behaviors that align with a given role in an interaction *and* when others act toward them in a way that sustains that role. Ineffectual performance or reception can result in *losing* face, at which point one’s identity in a social interaction becomes threatened, potentially producing affects typically labelled as “bad” such as shame, embarrassment, or anxiety (Goldsmith, 2007). The role of advice in the production of “bad” affects can be understood through the notions of face-threatening acts (FTAs), as described in Brown and Levinson’s (1987) politeness theory.

Brown and Levinson (1987) propose that the desire to honor and maintain face is a key reason behind the use of politeness or linguistic softening strategies in social encounters. The use of politeness is crucial to maintain face, as many social interactions can threaten face, and thus be classified as FTAs, including orders, requests, warnings, and *advice* (Goldsmith, 2007). In order to explore exactly how face is threatened in these social encounters, Brown and Levinson (1987) further categorize face as being either positive or negative. Positive face refers to the desire to have one’s image be recognized, accepted, and approved of by others. Negative face refers to the desire to have one’s autonomy respected, independence permitted, and to not be imposed upon by others. As an FTA, advice can be seen as jeopardizing both positive and negative face.

Advice giving as a practice “suggests that the advice recipient lacks knowledge or competence concerning the issue at hand or is unable to cope with a problem without external aid” (Feng and Magen, 2016, p. 752). By suggesting that the advisee is unable to act wisely on their own, notions of competence, value, and acceptability are challenged, threatening positive face (Goldsmith and MacGeorge, 2000). Similarly, advice giving by definition implies that the advice-giver has insight that the recipient lacks, “positioning the interactant asymmetrically” and potentially inducing notions of hierarchal valuation of both knowledge and self into the interaction (Feng and Magen, 2016).

Not only does advice giving threaten the recipient’s positive face, advice rejection is an FTA that can impact the positive face of the advice-giver. Advice rejection can be seen as a form of overt social rejection wherein not only is the advice rejected but, by extension, so too is the knowledge, value, and face of the advice-giver. Advice rejection can therefore be seen as symbolic of “an advisee’s devaluation of an advisor,” threatening their own understandings of their competence (Belkin and Kong, 2018, p. 181). Understanding the rejection of advice as a threatening of positive face and competence of the advice-giver is crucial, as Peluso et al. (2017, p. 501) suggest that giving advice “is one means to restore a sense of control” in one’s life and that it offers a means to restore that control because “it provides a signal of competence to an individual.” In a neoliberal western society, where the potential of disability itself is viewed as a deep threat to capitalism and control, giving advice to others operates as a means to assuage one’s own fears and reclaim perceived control over one’s own body. As such, a rejection of this advice is severe FTA, as the act of giving advice in the first place is means of claiming positive face in the form of competence signaling. Resultantly, the presence of an FTA on both sides of an interpersonal encounter can lead to heightened affective responses, further threatening face.

### 2.2 Shame and blame culture

It is important to contextualize the face-threatening nature of unsolicited advice within the broader context of contemporary neoliberal western society and how disabled people are articulated as objects of resentment within it—often acting as scapegoats for perceived societal ills (Hughes, 2015). Hughes argues that disabled people have been constructed under neoliberalism as synonymous with parasitism, fraud, and idle dependency—blameable subjects within what he terms a “blame culture” (Hughes, 2015, p. 993). I argue that within the context of unsolicited advice, disabled people emerge not only as blameable subjects but *shameable* ones. Such a conceptualization is indicated in the observation from Jóhannsdóttir et al. (2021, p. 354) that blame culture is “where shame is clumped and reinforced, and disabled people are even judged responsible for numerous societal problems.”

Theorizing shame sociologically, Scheff (2000, p. 96) asserts that shame is “a large family of emotions that includes many cognates and variants, most notably embarrassment, humiliation, and related feelings such as shyness, that involve reactions to rejection or feelings of failure or inadequacy.” The emphasis on rejection as a cause of shame is crucial in understanding shame sociologically, as it conceptualizes shame as resulting from a loss of social connection or a threat to the bond between oneself and another (Scheff, 2000; Bath, 2019). Understanding shame as always intra- *and* intersubjective, occurring in response to others, positions shame as “perhaps the most intimate of feelings,” as it can only be “brought into being by an intimate proximity to others (Probyn, 2004, pp. 330–331). For this reason, Scheff asserts that shame is “the premier social emotion” (Scheff, 2000, p. 84). Importantly, while shame is brought into being in the presence of others, the calling into being of shame also occurs in specific contexts and spaces and is inflected by historical and political circumstances (Probyn, 2005; Richards, 2019). Shame is a thus a complex entanglement of the personal, the political, and the social which constitutes “powerful material and discursive performances” (Shefer and Munt, 2019, p. 145). These performances of shame are instigated by and felt within the body as a “desire to ‘fit in’ and, at the same time, a feeling of being ‘out of place’” in space, context, and community (Probyn, 2005; Johnston, 2007, p. 30).

In the context of disability, shame as a feeling of “being out of place” is inherently intertwined with neoliberalism and the ways that disabled people’s mere presence can work against societal norms of self-sufficiency, meritocracy, and taken-for-granted independence. Here then, shame emerges from affective practices of shaming or blaming (such as unsolicited advice as an FTA), which themselves emerge from broader societal feelings of resentment toward disabled people. Indeed, Jonas identifies resentment as “an entry point for identifying the norms of advice giving” (2017, p. 815). In identifying the norms of advice giving, much pre-existing literature focuses on how to best give advice in order to minimize negative impact and experiences of rejection of the self (Hepburn and Potter, 2011; Jonas, 2017). Resultantly, the focus is moved away from the experience of the recipient of advice, with their affective response devalued in favor of the advice-giver. In this way, resentment does not end with the advice itself but extends beyond it into the reception of reaction. In contexts of advice with disabled people as the recipient, this may align with the abjectification of their identity, wherein their social worth is devalued and stigmatized, positioning them as “objects of disgust” (Hughes, 2015, p. 996).

This positioning may serve a powerful purpose in neoliberal advice transactions, as affective intensities such as disgust have been identified “as key strategies through which the neoliberal subject becomes engaged in the task of its own self-governance” (Parker and Pausé, 2019, p. 251). Thus, in a neoliberal context, the positioning of disabled people as objects of disgust within a ‘blame culture’ may be crucial to the navigation of unsolicited advice, as the simultaneous abjectification of disability identity and a collective societal resentment serves to devalue disabled individuals’ face. This devaluation of face may serve to minimize the collective responsibility in interactions to save face, decreasing the desire for politeness in navigating FTA and instead positioning such advice as deserved and in fact *necessary* for the restoration of the collective neoliberal order and individual notions of merit.

### 2.3 Against the shame/pride binary

While shame, blame, and resentment are often binarily constructed as purely “negative”—the antitheses to disability joy and pride—it is important to consider how these affects indicate an attunement to environment and connection and the ways that they are engaged and/or resisted. Literature on shame resistance as it relates to disability is typically articulated through the language of a journey *from* shame *to* pride, in overcoming, in passing through phases and acceptance processes, and ultimately in “arriving” at pride and self-recognition (Morris, 1991; Brown, 2003; Manessis, 2014; Richards, 2019). The language of the journey is present in articles and memoirs navigating disability shame/pride, with the beginning exploring the feelings of denial and shame that accompany the onset of or recognition of disability and concluding with a triumphant declaration of pride, shame long forgotten. While these narratives bring important first-person perspectives and explorations of shame/pride to the fore and articulate the experience of disabled pride in a critical way, the neat acceptance narrative that resolves with a triumphant overcoming comes with affective implications and material effects.

The linear trajectory of shame to pride in many ways mirrors linear notions of healing and development that reinscribe disabled people as deficient and continue to position a whole and normative self just out of reach. Such framings of pride as the natural endpoint of a disabled affective identity and experience create parallels of overcoming: one must overcome their impairment and shame for acceptance in the general population, personal life, and disabled community. The parallels of overcoming are reinforced by the medicalization of shame in disabled narratives and the move toward bio-psycho-social interventions into disabled lives to promote pride as a “protective factor for self-esteem” (Bogart et al., 2018, p. 155). The medicalization of shame thus works to discursively construct pride not as a radical or resistant act of self-reclamation but as an affect indicative of better psychological outcomes and alliance with normative mental well-being. While positive self-esteem is no doubt important, the construction of pride as a medical outcome that works to deny or mitigate shame positions it as another aspect of the self for disabled people to control. Thus, pride becomes another tool of medical responsibilization.

Pride, and in many cases joy, therefore become for disabled people an element of what Frye (1983) terms the affective double bind—wherein the oppressed are required to perform a degree of happiness and cheer. To be oppressed is therefore to also be expected to engage in an affective performance that upholds the fantasy of happiness and, in the case of disability, meritocracy and neoliberalism that the broader population is oriented to. Resultantly “anything but the sunniest countenance exposes [marginalized peoples] to being perceived as mean, bitter, angry or dangerous” (Frye, 1983, p. 2).

It is from this understanding of the double-bind of oppression, and potentially harmful implications of seemingly solely “positive” emotions such as pride, that an exploration of the positive or generative potentials of shame become clear. Ahmed (2010b, p. 67) identifies what she terms ‘affect aliens’ as those who are “affected in the wrong way by the right things” or who “affect others in the wrong way.” In the first sense, one may be affectively alien not necessarily due to responding to the same events or objects as others with the wrong affect (e.g., feeling joy when others are sad) but rather by experiencing an affect in relation to the what others deem “the wrong objects” or events (Ahmed, 2010b, p. 171). In the instance of disability, which is broadly recognized as an object of tragedy that should invoke affects of pity and sadness (Goodley et al., 2018), experiencing joy, pride, ambivalence, or any other affect thus results in a disorientation to the expected collective affect and renders one an affect alien. While this alienation can be isolating, Ahmed also indicates that affective alienation can work to expose the origins of violence and act as a form of consciousness raising (2010). Indeed, Ahmed asserts that “the act of noticing limitations can actually make life seem more rather than less limited” (Ahmed, 2010a, p. 584). In this way, seemingly negative emotions such as shame can in fact open up new ways of being in the world that acknowledge the role of oppression and move toward collective liberation. Some disability scholars and artists, including Clare (1999), Chandler (2009), and Chandler (2014) have spoken to the impossibility of the shame/pride binary, particularly as it relates to desire and belongingness in disability and queer communities. The explorations of these scholars form the foundation on which explorations of unsolicited advice and affect can be built. This includes the intertwined desirability of politicized identities, the pride with which we relate to them, and the shame that they can generate in simultaneity.

Thus, it is from these academic explorations, affective frictions, potentials, and sociopolitical contexts that this study and its guiding research questions arise. Very little literature on the reason for and the experiences of unsolicited advice for disabled people exists (For an example see Vayreda and Antaki, 2009); however, the prevalence of this social issue is indicated by the amount of non-academic articles, memes, art shows, and disabled cultural productions that speak to disabled people’s experiences of unsolicited advice (For an example see @unsolicited_advice_projects on Instagram). Using this conceptual framework built from a constellation of critical disability studies, affect theory, and extant literature on unsolicited advice, this research seeks to qualitatively explore the affective experiences and political implications of unsolicited advice.

## 3 Methods

I approach this work from my position as a white, queer, multiply disabled person who was raised by a disabled mother. This coalescing of identities critically informs the way that I have approached this research, its participants, and my engagement with the role of ‘researcher.’ I informed participants from the outset of my positionality, and it often further emerged in conversation throughout interviews. As such, I cannot lay claim to the role of the detached or ‘objective’ researcher but instead locate myself as deeply embedded in this process. I note this in order to account for and engage with reflexivity, both in the data collection process and in the restorying of my participants narratives. However, I do not wish to imply that my disability here is a disadvantage or threat to the integrity of the study; rather, I see it as my greatest strength. In recognizing qualitative interview spaces “as intersubjective emotional encounters” (Hoggart, 2021, p. 582) inherently imbued with personal values, I am able to utilize my own lived experience as a disabled person in navigating the *emotional* rapport of the research space in a way that is both informed by, and informs my use of, affect theory and narrative inquiry.

### 3.1 Participants and sampling

This research draws from interviews conducted with 15 disabled participants residing in Ontario, Canada. Due to the ongoing COVID-19 pandemic, as well as in an effort to increase access for potential participants, all recruitment and data collection for this study was conducted through online means. A non-probability convenience sample was initially collected via social media recruitment, with additional snowball sampling occurring as participants recruited their own social networks in response to their own interview experience. All participants for the study were required to meet the following sample criteria: (1) be 18 years of age or older; (2) reside in Ontario, Canada; (3) be able to communicate in either English or ASL; (4) identify as disabled; and (5) have received unsolicited advice about their disability or health more broadly.

Recruitment was undertaken with a goal of recruiting 12–15 participants for the study—a number that aligns with extant literature indicating that in-depth qualitative interview data typically reaches saturation within the first 12 interviews (Guest et al., 2006; Brian and Clarke, 2013). Between social media recruitment and participant referrals, a total of 24 individuals responded to the call for participation. Out of this initial sample, three participants were deemed ineligible as they did not meet sample criteria, and a further attrition of six participants occurred due to either a lack of monetary compensation or fluctuating capacity due to health considerations. As a result, the final sample of this study consists of 15 participants (*n* = 15). Demographic information along with pseudonyms chosen by each participant are outlined below^2^^,^^3^:

**Table 1:** 

**Pseudonym**	**Age**	**Pronouns**	**Racial identity**	**Disability type**
Adrian	30–39	He/Him	White	Neurological / chronic illness
Alexis	40–49	She/Her	White	Chronic illness / neurodivergence
Ayla	19–29	They/She	White	Cancer / chronic illness
Brooke	30–39	She/Her	White	Physical / Full-time wheelchair user
Eljay	60–69	He/Him	White	Physical
Helen	50–59	She/Her	Asian	Neurodivergence
Honey	19–29	He/They	White	Physical / neurodivergence / neuroqueer
Lily	19–29	She/Her	White	Neurodegenerative / autoimmune / physical and cognitive
Miki	60–69	She/Her	White	Cognitive
Reese	19–29	She/They	South Asian	Physical
Robin	19–29	They/Them	White	Chronic illness / neurodivergence / wheelchair user
Saff	30–39	They/Them	Mixed-race	Neurodivergence / chronic pain / developmental / mobility
Sam	19–29	They/Them	White	Neurodivergence / chronic illness / physical / mobility
Sara	40–49	She/Her	White	Neurological / mobility / neurodivergence
Toni	30–39	She/They	White	Physical / chronic pain / neurodivergence

### 3.2 Data collection and analysis

Data collection took the form of in-depth semi-structured qualitative interviews that took a narrative inquiry approach. Narrative inquiry, which approaches interviews and research with a deep and rich investment in participant stories, was selected due to its potential to intertwine storytelling, emotion, and theoretical inquiry such that it produces *lived theory*, connecting the “daily life of the protagonist” (participant) with broader social issues (Kim, 2008). Interviews were conducted over Zoom and ranged in length from 45 to 80 min. Participants provided written consent prior to booking an interview and verbal consent the day of the interview to ensure ongoing consent and mutual understanding. All interviews were transcribed verbatim to maintain the unique ways that participants spoke, in part due to their disabilities. As such, stuttering, stammering, and tangents were included in the final transcripts as data relevant to the participants at hand. In line with a grounded theory approach, data analysis was conducted through two rounds of inductive emergent coding using NVIVO. This grounded approach, which allows inductive theories and themes to be generated inductively *from* the data, was chosen due to its alignment with the inductive sensibilities of narrative inquiry. Further, due to the permeable and slippery nature of emotions, coming up with fully discrete categories of overarching affective response was avoided in favor of larger umbrella categories of an overarching affective ‘stem’ (e.g., anger, fear) with more specific terms and experiences articulated by participants used as subcodes.

Categories of affective response were grouped using Willcox’s (1982) model of the ‘feeling wheel’, in which language to describe emotive responses are grouped by “primary feeling,” those typically considered “primarily pleasant emotions” (peaceful, powerful, and joyful) and “those which are usually unpleasant” (sad, mad, and scared) (274). The feeling wheel model is useful for an affective examination of unsolicited advice over time, as the layout of the wheel includes the opposite correlate of an emotion where the supposed binary inverse of an emotion is included directly across the wheel. This, Willcox (1982) asserts, allows for a conceptualization of “the process of converting feelings” and the affective bridges that exist through coping mechanisms. This model therefore allows for an understanding of how the affective response to unsolicited advice may be converted over time. Using this list of thematic codes and the narrative arc that emerged through analysis, I then set about re-storying the collective participant narrative, slotting thematic codes into the narrative sections that they aligned with and generating a tentative timeline of affective experience. This timeline begins with initial affective responses such as fear, hope, anger, and shame. It then traces how these initial affective responses shift over time toward sadness, loneliness, and apathy in potentially empowering and resistant ways. This narrative and thematic list forms the outline of the following results section.

Ethics approval for this project was sought and received from the Queen’s University General Research Ethics Board (GREB).

## 4 Results

Throughout participant’s stories about their experience with unsolicited advice, a clear narrative chronology emerged that coincided with several key themes. Participants articulated the way that their internal affective response and outward social performance to unsolicited advice had changed over time. This varied based on disability onset and age, but overwhelmingly there was an articulation of a trajectory from initial experiences of unsolicited advice in adolescence or adulthood (upon disability onset) toward different ways of knowing and being in those interactions. This entailed a move from initial affective responses of *fear, hope*, *anger*, and *shame* in reaction to unsolicited advice toward *apathy,* which allowed for the negotiation and embrace of seemingly negative emotions such as shame and sadness. However, it is important to note that while this narrative arc was evident across all participant narratives, it is far from a linear trajectory. Even as participants described the onset of different emotions over time, others persisted or existed in tandem with those experienced initially. Thus, while a chronology of emotions is clear in the data, and is used to structure the following results, it is inherently complicated by the cyclicity and simultaneity of human affect.

### 4.1 Initial affective response

In telling their stories, participants indicated that their response to unsolicited advice initially, both in adolescence and upon disability onset, was a strong internal affective reaction. Importantly, however, this internal emotional response did not seem to align with an external performance in the social interaction, with participants instead indicating that they were less likely to “stand [their] ground” (Brooke), due to a more limited understanding of themselves as disabled people and what worked for their symptoms. This more limited understanding of themselves, as well as the newness of disability, meant that participants were experimenting with what felt okay to them. Toni discussed this experience in the first few years after disability onset:

I kind of had to go through this period of time where I was trying to figure out what my boundaries were, particularly around advice and suggestions and care. And I think a lot of people go through that because, initially, if it’s something you have never experienced, it’s scary, and you want it to stop, or you want to find solutions. You believe there might be solutions and you believe that those solutions would take the form of the health condition not existing. So, I think in that time I was a lot more vulnerable to the input of others and more open to it.

As participants discussed their perceptions of unsolicited advice, their affective response, too, shifted. Ayla noted that “your initial emotional response, adolescent emotional response is typically not very articulated” and therefore came with some strong emotions—emotions that Reese spoke to in their assertion that.

when I was first diagnosed, I kind of did feel some resentment. I thought like, look, I tried all of these things and they did not work. And I still have this like issue that I now have a name for… but none of these things actually helped. And you know, just being like an angsty sort of 20-year-old, I just like, I would kind of want to go off on these people and be like these things aren’t helping!

Beyond change over time, participants described their affective response to unsolicited advice in expansive and varying terms. While Willcox’s (1982) model of the feeling wheel, which guided the initial categorization of affective categories (see Methods), labels shame as a secondary feeling of sadness and hope as a secondary feeling of power, I have chosen here to explicitly name them as their own categories due to the prominence of both of them and their inverse correlate in participant narratives. Therefore, the most prominent initial affective responses to unsolicited advice can be described as *fear*, *hope*, *anger*, and *shame*.

#### 4.1.1 Fear

Fear was a prominent affective response animating participants’ discussions of unsolicited advice and disability. Participants used words such as unsafe, insecure, uncomfortable, triggered, dread, doubt, insecurity, anxiety, concern, confusion, helplessness, and rejection to describe the emotional response that unsolicited advice evoked. As previously discussed, the newness of disability, or of disability in adulthood, meant that participants described feeling confusion, anxiety, and fear about the progression of their disability as well as the social interactions they were now confronted with. For some, this fear and discomfort emerged from a lack of words to describe their experience. Lily described this, stating “when I first received that piece of advice, it made me uncomfortable inside, but I did not know how to verbalize how it made me uncomfortable, and so I kind of just took it.” For others, the fear came from a place of feeling like they were unable to “communicate to other people safely.”

This fear of being unable to safely communicate was grounded in unsolicited advice being perceived as (and sometimes explicitly working as) accusations of malingering, leading participants to question whether their actions and behaviors in relation to their disability were the “correct” ones. This self-doubt and anxiety were described by Toni and Honey:

Yeah, there was a time where I would leave those conversations [around unsolicited advice] feeling like maybe I’m not doing enough. Maybe I am making the wrong choices. Maybe I would be in better shape if I were doing things differently (Toni).

I do still have that experience of like, am I over exaggerating? […] I feel like a lot of the unsolicited advice, at least that I receive, stems a lot from like “you are overexaggerating” and like “things are not this bad,” and “you are just imagining it” (Honey).

Accusations of malingering, both explicitly made and implied by experiences of unsolicited advice, produced self-doubt, anxiety, and fear in participants who were made to question if they were doing enough. This impact of unsolicited advice was summed up by Reese as “very triggering for me, and makes me really like anxious… and I do not know it’s just… it feels overwhelming.” Ultimately, through lack of vocabulary and knowledge about disability, and a lack of safe space to communicate due to accusations of malingering, unsolicited advice worked to produce initially fearful and anxious reactions in participants.

#### 4.1.2 Hope

The uncertainty and fear that participants felt around their disability and unsolicited advice also lent itself to the potential onset of hope at the advice and opportunities being offered. Toni and Lily discussed the increased openness they felt to advice at the beginning of disability onset, due to fear with a desire to grasp “at anything that could possibly help” (Lily) because “it’s scary, and you want it to stop, or you want to find solutions” (Toni). Reese described their experience with the hope that unsolicited advice inspired in this context, saying “toward the beginning of my disability journey when these elders would kind of give this unsolicited advice it would kinda like, raise my hope a bit.” Honey spoke extensively to this idea of hope, recognizing that as someone who is newly diagnosed and therefore “recently new to disability” that unsolicited advice still gives them a sense of hope and excitement. He explained:

So, when I receive this unsolicited advice I get excited because I’m like this will finally work and like, especially when it’s newer unsolicited advice […] it’s like, it’s excitement! It’s like, oh, my gosh! I finally found something that might work.

However, this hope was complicated by cyclical feelings of disappointment that emerged when advice did not work, a disappointment that was heightened by the repetition of the hope cycle:

And then it loops back into when it does not work, then there’s something even worse. So, it’s kind of this, and this loop of like I feel really excited when I receive unsolicited advice that’s brand new, and then when it does not work and I hear it again, it turns into like this disappointment, and like it, kind of reminds me of that… like something… it feels like something is even worse than it was originally whenever I hear advice that’s been repeated over and over, just because, like, if I’ve tried it, and other people are recommending it, that means that it must have worked for them (Honey).

Through the affective cycle by which unsolicited advice inspired hope and then disappointment, this disappointment was slowly converted or ‘bridged’ (Willcox, 1982) into frustration. Honey articulates this in his discussion of frustration and hope coexisting: “I think I think there’s still that frustration there, but I think it comes across as this hope of like this, fresh like “Oh, my gosh, I gotta do this again.” But also, there’s this obviously new opportunity. Through this affective conversion articulated by the participants who experienced hope, the theme of frustration, or anger, emerges.

#### 4.1.3 Anger

Much like fear, anger was a dominant primary emotion in the affective narration of people’s experience with unsolicited advice. Participants described their anger using words like anger, ire, hostility, irritation, frustration, aggravation, annoyance, resentment, and betrayal. When asked what emotions unsolicited advice brought up in them, Brooke responded saying “that is pure frustration for me” or “sometimes, depending on the circumstance it could be a little anger too” while similarly others articulated unsolicited advice as producing “indignance, frustration, aggravation” (Miki), and largely making participants “fucking mad. It’s just sort of like, really?” (Sara). Much like the impetus for fear, experiences of anger too emerged from the accusations of malingering, lack of self-knowledge, and incompetence implied by unsolicited advice. Participants identified frustration, irritation, and anger as coming from “sort of like a feeling of being condescended to” (Ayla) and as triggered by assumptions that participants were faking “to avoid working, to you know, sponge off of society, you know? That stuff can be very angering” (Eljay). Robin discussed the implications of these assumptions more, saying.

like because I already feel like I am not good at like doing things, I do not feel like a capable person, it like triggers me to think that they are just being judgmental. You know what I mean? So, it instantly like pisses me off because I’m like you are just assuming, you know?

For Honey, unsolicited advice and its attendant assumptions were even more frustrating when they did not “come from like a place of care, and it just comes from like a place of fixing.” Conversely, in a medical setting, Saff highlighted the feeling of frustration and betrayal that can emerge when one is actively trying to find a solution and instead gets advice on an unrelated matter (for example, advice on weight loss when seeking help for chronic migraines). Saff stated that unsolicited advice “when it’s from a medical professional, it’s betrayal. Yeah, because these are the people that we go to for help, because hey, I’m in pain.” Here, non-disabled people’s self-assigned expertise in disabled persons’ wellbeing and the role of the “cloak of incompetence” are highlighted across both non-medical and medical settings.

Unlike fear but similarly to hope, repetition of unsolicited advice played a role in the affective response of anger, often articulated as frustration or annoyance. Participants described being aggravated by the repetition of advice that further assumed their incompetence and pulled them back into an unwanted social interaction.

#### 4.1.4 Shame

Participants also consistently highlighted the role of unsolicited advice in producing shame. Participants both explicitly named shame and alluded to it through continually identifying self-consciousness, self-loathing, embarrassment, rejection, and inadequacy. This aligns with Scheff’s (2000) sociological theory of shame that aligns shame with embarrassment, humiliation, rejection, and feelings of inadequacy. In describing their own emotional response of shame, participants also pointed to unsolicited advice as “a moral shaming” (Alexis) that was felt most deeply “at the beginning” (Sara) of one’s disability journey. This presence of feelings of inadequacy in initial experiences was articulated both by participants with adult-onset disabilities, like Sara, and in adolescence. Ayla spoke to this, saying that in their adolescence “there was a very strong sense of like self-loathing.”

Participants named that unsolicited advice caused “all those thoughts of self-doubt and inadequacy” (Reese) that “hurt because it’s like oh, well, I’m never going to be enough” (Saff). That shame is felt within the body as a “desire to ‘fit in’ and at the same time as a feeling of being ‘out of place’” (Johnston, 2007, p. 30); this was described by participants who discussed shifting their behaviors in an attempt to mitigate shame. Helen offered one such example:

And so something [embarrassing] like [an awkward interaction in the hallway] happens, and people start thinking you are weird. And then, because, you know, people think you are weird, you start being really self-conscious, and maybe behaving weird, or you know, behaving differently, like avoiding people, going down different hallways and things, and it just sort of built to a point where people may get burnt out, or they might have a meltdown, or, you know, be in some kind of real distress.

Here, Helen points to the experience of shame pushing her to shift her behavior in order to avoid other shameful experiences. This reflects Probyn’s (2004) assertion that shame is incorporated into how one moves in the world. This further aligns with Tabin et al. (2019), who asserted that shame emerges through loss of connection and rejection by others. While Helen here highlights that loss of connection and subsequent avoidance, Sam spoke at length to the role of unsolicited advice in causing feelings of rejection:

Yeah, again, I think rejection. Is that an emotion, that sense of rejection? […] I think that for a lot of people, and probably myself included unsolicited advice, I think, triggers rejection sensitivity in that people immediately feel or can feel that the advice, because again, the perception of what advice is going to vary. But people perceive it as an attack on them, their character, their experience, whatever and then, in response, become defensive.

The rejection, shame, and sense of being attacked that Sam identifies here in the action of unsolicited advice connects to the idea of shaming as an affective practice that works to produce shame as not only an emotion but “a moral tool” (Tabin et al., 2019:90). While participants were encouraged to think broadly about who gave them advice, and no parameters on the kind of advice-givers they could talk about were given, participants exclusively gave examples of advice from non-disabled advice-givers, suggesting the weaponization of shame as a moral tool by the non-disabled populus specifically.

This was just one way that unsolicited advice as an affective practice produced shame, with other participants identifying interlinked practices of mockery, labelling of burden, and assignation of moral blame. Lily spoke at length to the ways that unsolicited advice worked to produce shame:

Anyways… burden, shame, of course. How could it not? If someone says, hide exactly this thing from me, how could that not make me feel shame about it? You know it like so blatantly communicates that they do not want to see that part of me, or that they do not want that particular thing to happen to me […] Yeah, for sure, it definitely makes me feel like they, they interpret me not following their advice as an opportunity for me to become more burdensome. And then that it is shameful that I would not take their advice, because you know, they are giving me a nugget of wisdom that will allow me to, you know, maintain goodness in their eyes, you know?

This idea of needing to take on and comply with unsolicited advice as a way to mitigate symptoms or reduce burden on others—a burden that is identified as a moral failing—was highlighted throughout other participants’ stories too. Miki highlighted that unsolicited advice sometimes worked to establish her as having caused her disability herself, saying “it’s that whole fatalistic, either I invited it, or some force intended it to happen to me. But what they mean when they say that, like if I were really to absorb that I would be living with guilt, with the idea that I’ve done myself damage.” Ayla too identified that with unsolicited advice “if you fail to like, do any of these things that people are suggesting it’s sort of like bringing your death upon yourself,” further asserting that it is a way to create a moral blame or find a fault as to why a condition occurs. The notion of fault was echoed by Brooke, who spoke to an intertwined experience of disablist and fatphobic shaming at a medical clinic:

That nurse that that shamed me at that clinic… it did feel like shaming. It did feel like fat shaming. It was like my fault I was obese, and like one, I’m a wheelchair-user I have no mobility in my legs at all and so like exercise is difficult. You’re not going to find me at the gym six days a week [Laughing].

Ultimately, participants’ stories pointed to the ways that unsolicited advice operated as an affective practice that worked to position disabled people as responsible for risk mitigation of their disability and to shame them into what advice-givers deemed morally “good” behavior. This aligns with the conceptualization of the moral economy, which a participant, Saff, further identified in their discussion that “we do live in that culture of shame.” Here, unsolicited advice thus operated as a tool to maintain belief in “a just world” where “good things happen to good people” and bad things happened to bad or irresponsible people who “deserve it” (Saff).

### 4.2 Affective response to advice over time

In narrating their experiences with unsolicited advice, participants described the shift that happened over time as they came to develop response scripts, coping mechanisms, and simply trust themselves and the communities that they found through taking their identities and disabilities seriously. Continuing with Willcox’s (1982) feeling wheel, this section explores how the experiences of participants with unsolicited advice—while in some ways co-existing with the four initial affective responses—largely shifted through sadness and loneliness toward apathy. While Willcox identifies *sadness* as a primary emotion, I pull out two of the secondary and tertiary emotions identified in more depth: *loneliness* and *apathy*. These affects were also held in tension with others, bridged or converted, and navigated through as varying resistance strategies.

Participants consistently described their reaction to unsolicited advice as shifting over the years, a process that was highlighted in particular by participants who had been living with their disabilities for a decade or more, as well as participants in their thirties and older. While all participants indicated a shift in response over time through their narrativization and anecdotes, these participants with decades of experience were quick to explicitly name the way that their experience had shifted over time and reflect on it. Toni asserted that their “reaction to [unsolicited advice] has changed a lot over the years,” a process that other participants described as consisting of both shifting internal affective reactions and development of external responses. Brooke spoke to this, explaining.

I had to learn over the years kind of how to stand my ground, and you know kind of navigate… and it has not always been successful. There have been, you know upset providers or upset people. I’ve been upset. It depends, you know, depending on the circumstances, but I do find that I’m getting… because it unfortunately repeatedly happens, I’m getting better at the response. I’ve kind of dialed in on how to respond.

The emotional element described by Brooke here was echoed by Eljay, who described how over his years of experience he has “been more inclined to react one way then another, more inclined to take it in stride and try to understand.” He further explained how around 5 years after disability onset, he was more prone to react with anger, but as time has gone his emotional reaction varies with mood, but he is more likely to “just let it flow […] like water off a duck’s back.”

Participants also echoed Brooke’s sentiment of dialing “in on how to respond.” Miki explained that “after 18 years you learn how to respond like you… you get the phrases, and if they dismiss you, you are willing, I guess, to dismiss them. Not them, but the comment.” This move toward internal dismissal of unsolicited advice was a prominent theme across participant narratives; however, it was not always reflected in the outward response within the social interactions. In addition to shifting affective response, these themes of dismissal of unsolicited advice and outward performances are discussed further below.

#### 4.2.1 Sadness: “at this point it’s more like existential crisis sadness”

Experiences of sadness and alienation dominated the continued affective narratives of participants, alternately described using words such as sadness, grief, hopelessness, depression, collapse, loneliness, disconnection, alienation, isolation, exhaustion, tiredness, and resignation. While this affective predomination of sadness guides the narrativization of this section, sadness continued to exist in tension with the other affective responses previously described. In particular, anger, most frequently described in the form of frustration and annoyance, continued to make an ongoing appearance; however, these affective responses of frustration also seemed to affectively bridge toward resignation and apathy, with the ongoing repetition of unsolicited advice providing the fuel for this emotional conversion. Frustration also emerged continuously as the trajectory from those who had initially experienced its inverse correlate, hope, and which eventually transformed into disappointment through storytelling. Therefore, while I take sadness as the primary emotion of interest here due to its narrative dominance, I do not wish to suggest that this dominance precludes other affective responses to unsolicited advice, nor that it exists without tension being held between it and other coexisting emotions.

Participants described how sadness emerged in their ongoing experiences with unsolicited advice as they came to realize, over repeated interactions, the critical ideological and social disconnection between them and the disablist society at large. Saff described how this impacted them, saying that the “knowledge that we could die and no one would really care, we are entirely disposable… that weighs on you.” For participants, this conceptualization of their disposability was manifest in unsolicited advice with the suggestions of ways to mitigate or “fix” their disability, representing a fundamental devaluation of them as people. Toni explained this, saying.

[disability] is beyond a specific bodily concern, it is your whole world, and I think that that is just so deeply misunderstood. So, it’s like they want it to be eliminated. But then it feels like they want you to be eliminated, like that’s what it becomes, because there is no separation for so many of us.

This recognition of the devaluation of disabled bodies led to a sadness, not necessarily rooted in shame or self-consciousness at one’s own disposability, but a broader sadness at the disablist state of the world and the impacts of oppression on themselves and others. Saff described being “unable to get over the injustice of that […] so, what ends up happening for me a least, is that it sends me into a place of collapse and depression.” This was echoed by Toni who described the way that unsolicited advice contributed to a feeling of “existential crisis sadness.”

#### 4.2.2 Loneliness: unsolicited advice as disconnection

Participants highlighted the role that unsolicited advice played in producing a sense of disconnection from others in social situations or heightening their awareness of relational disconnections that already existed prior to advice-giving interactions between themselves and others based on the advice-giver’s perception of their disability. Unsolicited advice was described as a moment in which the disconnect between the self and the advice-giver became clear, resulting in participants describing feeling disconnected, alienated, isolated, lonely, dismissed, and not seen, heard, or recognized as themselves. This was primarily described in relation to pre-existing relationships, and therefore was a moment in which ableist preconceptions of the participant, or broader experiences of “othering” that facilitated the interaction, became clear. For some participants, this disconnect between themselves and others felt so wide that advice-givers were described as “liv[ing] in another world” (Miki) that separated the two in the interaction. Lily spoke poignantly to this disconnect:

I think a lot of the time, especially when people are giving me unsolicited advice in the context of disability it highlights the ways that they feel disconnected from me, and that’s their way of communicating that. And a lot of the time it kind of like comes out of left field, like you do not really realize that that was a disconnect that you had in that relationship until they verbalize it through advice that they are giving.

This eerie feeling of someone not really knowing who they were was further compounded by participants’ description of unsolicited advice as a “dismissive” (Saff) action, which ultimately “bypasses the reality of [their] experience” (Toni). Helen spoke to the way that unsolicited advice worked as a dismissive strategy to produce disconnect:

But if you say like, “you know, you just need the right planner,” then you are sort of shutting that conversation down. You’re making them think that, you know, they cannot really confide in you, because you’ll just tell them what they should be doing, instead of listening.

In this way, unsolicited advice worked to not just make evident the presence of a disconnect between advice-giver and recipient but to cut off potential futures of connected interaction. This ongoing disconnection was described by participants as resulting in almost scripted behavior from advice-givers that relied on formality and an emotional detachment from the recipient that was seen as indicative of a broader social detachment from disability. Helen spoke to this, saying “people, have gotten to the point where they are dealing so formally with me now, and it’s like breaking my heart.” Unsolicited advice therefore caused disconnection or made participants aware of a pre-existing disconnection—a disconnection that was not temporary but sustained through ongoing alienation of non-disabled recipients through formal language and the repetition of that advice (discussed further below). Understanding this shift away from the initial highly intense and reactive affective response to unsolicited advice and toward a deeper societally oriented sadness is crucial to understanding how this disconnect produced a sense of exhaustion in participants that oriented them away from the advice-giver and toward apathy, resignation, and indifference.

#### 4.2.3 Apathy: the politics of disconnect

Participants repeatedly described how repetition of unsolicited advice and the resultant cycle of awareness of disconnect played a role in a move toward apathy, as they slowly resigned themselves to the experience of the interaction. Robin described how “it’s happened so so much like my entire life. Like anytime someone starts saying “Have you tried…?” I’m like, shut the fuck up, you know? Like do not start, please.” Similarly, Reese described how their current “initial reaction is usually like, “oh my God! This again?!” This process of dismissal, wherein participants recognized the cycle of unsolicited advice as an irritant to be dismissed, was described by Brooke who said “for someone whose had a lifelong disability, it’s like at this point you are not being helpful, you are just being annoying” and Adrian, who stated that with unsolicited advice “I already know that. Like I do not need to hear it, it gets to be annoying.”

To this irritant of unsolicited advice, participants therefore came to resign themselves to the situation. This was described by Adrian who said, “pretty much like there’s no point in disagreeing with them” and Helen’s statement that “I have to kind of resign myself to the fact that I’m an unwitting participant in the education of people.” Through this resignation to the experience of unsolicited advice, participants described how some unsolicited advice, typically the most often cited lifestyle-oriented advice, slowly came to affect them less. For Helen, unsolicited advice became “truly just words, and I’m like… okay?” which was echoed by Reese in their statement that unsolicited advice was “still not exactly welcome, but I do not dread it the same way I used to.” As participants came to dismiss unsolicited advice as just unwelcome words, they described a move toward apathy, where the feelings “just roll through [their] body” (Sam) and eventually “one day someone may say something and I just let it, you know, like water off a duck’s back” (Eljay).

This move toward viewing unsolicited advice with a degree of apathy was crucial, as it allowed disabled participants to affectively and effectively navigate the interpersonal dynamics around unsolicited advice. This move toward apathy can be seen as a resistant strategy to the harms of unsolicited advice as an FTA. The harm of unsolicited advice relies on the recipient of unsolicited advice buying in to the collaborative nature of the encounter wherein both participants attempt to save face and sustain the other’s as well. By becoming aware of the disconnect between themselves and the advice-giver, participants were able to affectively distance themselves from the collaborative nature of the encounter and the emotional impacts of attempting to maintain face in an inherently face-threatening situation. As the repetition of unsolicited advice was frequently contradictory (e.g., ‘you should go running’, ‘you definitely should not go running’), participants were able to dismiss unsolicited advice while simultaneously recognizing that any course of action they took would ultimately cause them to ‘lose face’ in the eyes of advisors.

This resignation to losing face was ultimately described as liberating by Toni, who offered that “in some ways that realization can be really freeing. Because once you realize that you are never going to get it right, then you do not have to try.” This liberation from resignation was compounded by an indifference and apathy to advice as participants came to dull to it through repetition. As the FTA of advice is heightened by any degree of obligation to follow the advice or a sense that taking the advice may constrain autonomy, by dismissing unsolicited advice as “truly just words” (Helen) that they were not obligated to follow, participants preserved their internal sense of negative face. In this way, participants resisted not only the internalization of negative face but also the sense of expectation to provide a smooth social interaction for those threatening their face. This simultaneous internal preservation of negative face and resignation to losing their positive face therefore worked to resist some of the affective modalities of emotions such as shame and fear.

## 5 Discussion: feeling ashamed and a crip politics of shame

While navigating incredibly different life circumstances, diagnoses, relationships, and contexts, participants collectively told a story of affective changes, wherein initial experiences of unsolicited advice brought about fear, anger, shame, and hope, which was bridged and converted over time toward affects stemming from sadness, notably loneliness and apathy. While there was a distinct shift toward different affective responses over time, it is crucial to note that these emotions continued to coexist, with initial responses not necessarily disappearing but merely becoming less prominent in participant’s stories about themselves and the world. This shift toward apathy and resignation as advice repetition caused it to lose its salience and allowed for participants to in some ways detach from the encounter of unsolicited advice as a face-threatening action. This ‘bridging’ of emotions opened up space for participants to resist the expectations of compliance or gratitude for unsolicited advice that they saw coming from advice-givers in an interaction and to also hold space for multiple emotions at once. Notably, *shame* continually emerged across all narratives as a crucial piece of the affective puzzle—an emotion that participants both continually made space for in themselves and saw as a direct process of *shaming* from some individuals giving advice.

The specifics of how advice operated as an affective shaming practice were deeply influenced by the specifics of relational norms between advice-giver and recipient and whether a disability was hidden or perceivable. Participants with hidden disabilities, such as neurological conditions, neurodiversity, or chronic illness, described most of the advice they received as coming from those who had reason to know their disability status, namely family, friends, coworkers, and medical professionals. Conversely, participants with perceivable disabilities, such as wheelchair users or individuals with other visible mobility aids or assistive devices, spoke more frequently to the role of unsolicited advice from strangers.

While unsolicited advice from all people worked as a moral tool, advice from those that participants were close to, such as family and friends, was often seen as intended with care, even if the impact was not experienced as such. Despite ‘good intentions’ (a term used frequently by participants) and an ethic of care, this advice was still perceived as a moral tool to restore them to a state of disablist and neoliberal conformity and often to soothe the advice-giver’s own discomfort or fear of someone they cared about veering from the path of normativity. Conversely, advice given by coworkers, medical professionals, or strangers was seen more directly as an attempt to “fix” the disabled person or eliminate the “problem” of disability altogether due to a socio-cultural devaluation of disability. Crucially, the relational aspects and perceivability of disability also impacted the perceived motivation for advice, with those with hidden disabilities seemingly more likely to be accused of malingering, whereas those with perceivable disabilities seemed more likely to be labelled as a burden.

Ultimately, in describing their initial affective experiences, participants identified unsolicited advice as an affective shaming practice that worked to reaffirm the moral economy in which disabled people were both “good to mistreat and good to be good to” (Hughes, 2012, p. 832). Here, unsolicited advice emerges out of what Saff identified as a “culture of shame” and what extant literature labels as “blame culture” (Hughes, 2015) where disabled people are subject to shaming due to the misdirected ideological rancor of resentment experienced by the non-disabled population for disabled people’s perceived production of burden. Importantly here, resentment and shame do not just appear but are institutionalized within a sociopolitical context (Mulligan and Brunson, 2020) that accounts for unsolicited advice’s presence across various interpersonal encounters, including clinical ones. Crucially, while unsolicited advice operates as a shaming practice, not all participants indicated that they had been a/shamed (Kolarova, 2012), pointing toward an uneven distribution of affective responses to shaming practices and the potential of resistance.

Beyond the uneven distribution and experience of shame in relation to shaming practices, the politics of affect here emerge in that, while non-disabled advice-givers are permitted to engage in shaming practices, disabled recipients of advice are expected to signal their docility and cheerfulness in the face of oppression (Frye, 1983). This is described in Reese’s prior assertion that the most frustrating element of unsolicited advice “is that you cannot really voice discontent about that, because people take it personally.” Here, disabled people are expected to be docile and tolerant despite Scheff’s (2000) assertion that a shaming practice does not need to be very strong to produce shame and Kolarova’s (2012) assertion that processes of shaming induce strong affective reactions. Thus, despite the likelihood of experiencing a stronger affective reaction than the potential discomfort being expressed through advice giving, disabled people must control their emotions or risk “being perceived as mean, bitter, angry or dangerous” (Frye, 1983, p. 2). This again represented a devaluation of disabled peoples ‘face’ needs in interactions, justifying FTAs such as unsolicited advice. The need to maintain docility is further exacerbated by intersections with other identities such as one’s gender, race, or class. Saff, a mixed-race AFAB non-binary person with a history of being coercively feminized, spoke to this, saying.

you cannot really have that emotional reaction because then you are going to be labelled as “crazy” in air quotes, more reactive, and that’ll be used against you. Oh, classic. And of course, they’ll rely on your intersections, so you are just an angry woman, you are just an angry like insert racial slur here.

Here, unsolicited advice works as a moral tool to maintain the colonial racist, sexist, disablist and cisheteronormative neoliberal status quo that relies on the production of the self as the “right” kind of person in order to achieve respect and be seen as morally “good.”

Beyond the obvious social and psychological impacts of unsolicited advice on recipients as described through these affective responses, unsolicited advice was also articulated by participants as causing direct material harm through access to resources and medical treatment. Participants identified that advice-givers operated under the assumptions that “well, this might help and if not, you know, it cannot hurt” but articulated that unsolicited advice, especially in the form of inaccurate medical information from doctors and others, “can hurt!” (Alexis). For this reason, unsolicited advice was sometimes “problematic, sometimes even dangerous” (Toni) as it impacted how and when participants accessed medical care (Alexis, Ayla, Brooke, Honey, Miki), increased their mental burden (Ayla, Helen, Reese), impacted career decisions and their initial capacity to identify with disability (Lily, Toni), and, in the case of participants such as Alexis, Lily, Miki, Saff, Sam, and Miki, actively impacted their disability symptoms and diagnostic experiences. Unsolicited advice can therefore be understood as acting as an affective moral tool with very real emotional, social, psychological, and material consequences. Resultantly, as outlined, participants described varying resistant strategies to mitigate these harms that resulted in affective changes over time, including resigning themselves to situations, joking around, dismissal, or setting explicit boundaries. Resistant strategies can be understood as any behavior, internal or external, that allows an individual to mitigate the potential harm of an affective shaming or blaming practice and/or which expands their feelings of agency and self-trust in social interactions, despite negative impacts.

Importantly, though I have outlined unsolicited advice as an affective practice and a shaming tool with incredible emotional and material consequences, participants did not describe in their narratives a full rejection of shame nor a complete embrace of disability pride at all times. Rather, participants were intentional in their narration, maintaining that both seemingly “bad” and “good” emotions coexisted across social encounters. This coexistence is crucial to acknowledge, as extant literature on shame and shaming practices has largely outlined resistant strategies as relying on an utter refusal of shame and a linear trajectory toward pride. However, this goal of pride as the telos of affective achievement and the linked refusal of shame does not just require cutting off shame itself but also requires cutting off interest. Shame as a relational affective practice is “reliant on the investment, interest, and attachment of the person being shamed” (Parker and Pausé, 2019, p. 255). Without interest, “there cannot be shame” and thus you cannot be ashamed of something you do not care about (Probyn, 2004, p. 329). To fully reject or transcend shame to pride, then, requires cutting off connection: to people, to worlds, and to futures. While such connections, or interests, open one up to shame, such connections and their attendant are part of an attunement to the social environment and others that are implicated in ways of being in the world and the productive potentials that can result from connection. Indeed, shame can be an indicator of a fraying or severed connection, helping to establish where and with whom we should invest time, interest, and care in rebuilding (Shefer and Munt, 2019).

It is here that one can connect the initial affective response of fear and shame to unsolicited advice with the disconnection that participants identified as they came to live with disability and unsolicited advice. Participants, rather than navigating a linear trajectory from this initial affective response toward pride, cutting off connection, instead spoke continually of an orientation toward connection that required sitting with their feelings, even “bad” affects, and allowing those emotions to guide them toward other people and other futures. In this orientation toward connection, participants therefore moved not from shame, fear, and anger toward pride but instead apathy and sadness. These affects, while typically considered “bad” or entirely negative, in the case of participant experiences of unsolicited advice therefore indicated an ongoing strength, determination, and choice to orient toward connection and community despite the double bind of oppression. They represented a choice that produced self-competence, community identification, and moments of connection and understanding that may have otherwise been lost. Thus, while unsolicited advice undoubtedly caused negative affective experiences, harm, and oppression, this did not exist in a vacuum and coexisted with resilience, resistance, and a desire for otherwise.

It is these open potentials that Tabin et al. (2019, p. 100) articulate in their discussion of shame being “not merely a negative emotion, the antonym of which would be pride,” but an emotion that both makes and is made of us, such that it “actively participates in the creation of the social world” (Despret, 2005, p. 246 translation cited in Tabin et al., 2019, p. 100). While shame was only one of many affects described by participants, shame, and the majority of other emotions discussed in this research, fall under the broader category of sadness on Willcox’s (1982) feeling wheel. These so-called “negative” affects can therefore also be understood as participating in the creation of the social world. Further, as unsolicited advice works as a tool of shame, regardless of whether shame may be felt, the social interactions that are induced by the potentialities of shame deserve attending to.

Understandings of shame as holding positive or productive potentials are well articulated by feminist scholars, who have articulated a feminist shame theory and feminist politics of shame (Fischer, 2018; Shefer and Munt, 2019). Probyn conceptualizes shame as politically productive and as useful to the project of social justice in its capacity to advance a “project of everyday ethics” (Probyn, 2004, p. 329) and “develop a wider notion of the everyday - of what is personal and what is social” (Probyn, 2004, p. 336). Shame’s capacity to add intensity and interest to experience is also argued to hold productive potentials through its incitement to re-evaluate behaviors, perceptions, or connections (Probyn, 2005; Richards, 2019). Shame thus offers a “powerful resource for social critique” in its embodied relationality, forcing one to consider their connections with others and what interest, what frayed connection, the shame derives from (Shefer and Munt, 2019, p. 152). This role of shame in social critique was articulated by Toni:

there maybe was a time where it was my shame. But at this point it’s [advice-givers’] shame, and that’s what makes it particularly foul to me in my life now so I do not feel as threatened by it, but I just feel like… why are you putting that on me? Like you have tons of work to do go, do your work over there.

The power of shame to compel inspection of daily lives and what lives are made available and to whom thus acts as a catalyst for “an ethics of the everyday (Shefer and Munt, 2019, p. 151).

Shame’s “call to action” (Richards, 2019, p. 271) has been taken up by queer theorists such as Munt (2007) in their exploration of the shame/pride divide, with the emerging question being not ‘how do we resist shame?’ but rather “what will we do with our shame?” (Johnston, 2007, p. 37) The question of what shame might become, or the potentialities of affects so reliant on mutual investment, point to the ways that so-called “bad” affects might instead move us toward alternative futures. By entangling affects, temporalities, and narratives and challenging the notion of a linear trajectory away from “bad” affects and toward “good” ones, I argue that this research plays a role in cripping the politics of emotion. Just as queer theorists have articulated both queerness and affect as things to be queered, crip theorists, such as Kafer (2013), gesture toward crip as a way to destabilize conceptualizations of disability and disabled identity. With unsettling affinities “[c]rip and queer mark out, and indeed, flaunt the failures of normativity” and work to embrace “the possibility of an outside or more-than-one” (Fritsch and McGuire, 2018, p. i). It is this notion of crip as embracing the more-than-one that indicates a need to move beyond the binaries of shame/pride, good/bad emotion, and hope/apathy and toward an understanding of these emotions as affectively entangled and immersed in a broader blame culture.

A crip politics of emotion sees shame and other “bad” emotions not only as holding productive potentialities through its appeal to socio-emotional connection but as inherently entangled in the politics of pride, hope, apathy, and resentment, amongst others. As the linear notion of the shame-to-pride journey requires the refusal of shame and the positioning of oneself as the privileged exception within blame culture, pride, as it is usually conceived of, mirrors “disability shame: a shame construed by the very logic of conditionally tolerated exception” (Kolarova, 2012, p. 266). As such, a simplistic understanding of pride, or other “good” emotions, as resistance to shaming practices does not offer the keys to disabled liberation. Pride here is a closed future, limited in its potentiality. A crip politics of shame understands shame and pride, joy, hope, apathy, etc. as always co-existing, dynamic, and in tension. While the affective intensities of all may vary, these coexisting emotions work to map out the political horizon—“political imaginaries and their conditions of possibility” (Gould, 2009, p. 262). The affective intensities and practices of shame and pride work together to map out relationalities, indicating which connections are strong and which are frayed. A crip politics of shame understands shame, and the strategic performance of shame itself, as part of the survival kit of disabled people, with the persistent attunement to the environment indicating which connectivities are safe and which are not.

Beyond indicating what connectivities are available and safe, a crip politics of emotions understands affects such as shame as occurring not from an inability to ‘fit in’ to a societal mold or overcoming of said mold but from resentment structures such as unsolicited advice that construct the disabled subject as a/shamed. Affects thus cannot be transgressed by an individual in a linear path toward other ones, as the process of becoming a/shamed, and the experience of encounters such as unsolicited advice, are triggered by one’s existence within the broader label of disabled. Moving toward a crip politics of emotion means accounting for the varied affective intensities of both “good” and “bad” emotions, understanding that affects indicate political horizons and, indeed, the crip horizon. Not only do the affective practices of resentment, blame, and neoliberal shame structure worlds, but the coexistence of affective experiences respond in a structuring way. By accounting for the political capacity of “bad” affects in disabled experience, there opens up potential to understand disabled experience beyond linear narratives. Such potentials have the capacity to disrupt affective understandings of disability and resist the structures of resentment. Through embracing “bad” emotions and taking “good” emotions off their pedestal, the structures of resentment, while affectively intense, lose their assimilatory powers of “cultivating subjects “in the right way”” (Ahmed, 2010b, p. 32). Ultimately, by embracing the coexistence of affects, of narratives, and of resistant, reproductive, and shaming practices, ways of being otherwise are made clear.

## 6 Conclusion

This research sought to understand the affective impacts of unsolicited advice on disabled people and how they may negotiate and resist the emotional impact of these experiences. Despite varied experiences, backgrounds, and disabilities, participants articulated similar, though not linear, affective arcs in their narration. They spoke to the ways that, while unsolicited advice acted as an affective shaming practice and moral tool that caused direct psychological, emotional, and material harms, that their emotional response resisted easy categorization into shame or transcendence of so-called “bad affects” toward pride and happiness. Instead, participants described an enduring relationship with shame and other seemingly “bad affects” in a way that allowed them to move toward apathy and an engagement with a broad spectrum of emotions. In this way, participants not only resisted the shaming practice of unsolicited advice but also resisted the individualizing narrative of overcoming, so often present in disability narratives, that argues for a linear trajectory from disability shame to disability pride. Participants thus engaged in a crip politics of emotion, and specifically a *crip politics of shame*, that allowed new ways of being in the world that refused both narratives of vulnerability and of overcoming, inspiration, and pride, allowing them to instead just *be*. Here, a *crip politics of apathy* becomes crucial to understanding and reimagining shame, and crip shame, as an affect that can exist in the grey zone of affective intensity, compelling one neither to pure shame nor pure pride. Crip apathy allows for a rejection of *shaming* without a rejection of shame itself, moving beyond the binary of shame/pride, good/bad affects, and shame/shaming. Thus, to understand crip shame, one must understand crip apathy, against the backdrop of other “bad” affects, as decreasing the usefulness of the “tool” of non-disabled shaming itself.

In doing this research I wish to reiterate that participant’s narratives at times disagreed with each other, indicated different ways of knowing and being in the world, and are informed by my own affective experience and narration of their stories. I attend here to the ways that this qualitative research inherently relies on complex personhood wherein “the stories people tell about themselves, about their troubles, about their social worlds, and about their societies’ problems are entangled and weave between what is immediately available as a story and what their imaginations are reaching toward” (Gordon, 1997, p. 4). Thus, the stories that are told here do not represent a homogenous story of disabled life or experience and, while at times explicitly reach for the desired futures and interactions otherwise, are also at their core informed by what participant’s desires and imaginations, apathy, and resistance are gesturing toward. These desires coexist with the oppression articulated by participants in their narratives and across the page. This coexistence is crucial as, as a theoretical concept, “desire interrupts the binary of reproduction versus resistance” wherein it is believed that “people are bound to reproduce or replicate social inequity or, on the flip side, that they can resist unequal social conditions” (Tuck, 2009, p. 11). Rather, this research seeks to demonstrate that resistance can look like the reproduction of social inequality in the double bind of oppression and, conversely, that apparently resistant actions can instead work to individuate the resistor as a privileged exception and reaffirm oppressive ideals. As I have argued throughout this work, there is a need therefore to not only allow desire and damage to coexist in narrative space but to move away from the binary and linear assumptions of emotional trajectories.

## Data Availability

The datasets presented in this article are not readily available because ethics approval and participant consent forms both indicated that only the researcher would have access to the raw data. Requests to access the datasets should be directed to Megan Ingram, megan.ingram@queensu.ca.

## Footnotes

^1^I use identity-first language as opposed to person-first (e.g., persons with a disability) in alignment with disability justice activists and scholars as well as out of my own preference as a multiply disabled person.

^2^Pronouns are used in lieu of sex or gender categories based on the desire of several participants to have their specific gender identity or sex assigned at birth remain unclear to the reader in the write-up of the research. However, to respect the co-existing desire of some participants to speak to their experience as trans folks or as “women, femmes, or coercively feminized people” (Kayn, as cited in Ratchford, 2019), data about specific participants’ self-identification is provided as relevant throughout this article.

^3^Disability “Type” does not suggest an official typology but rather reflects the language chosen by participants specifically to self-describe their experiences in and with their bodies. This choice to opt for self-description is grounded in a desire to restore autonomy to disabled people in the research process and a refusal to collapse disabled experience into researcher-selected discrete categories. As the *affective* experience of disability is what is at stake in this research, the terms that brought participants the most *comfort* were prioritized.
